# G-Quadruplex Modulation of SP1 Functional Binding Sites at the *KIT* Proximal Promoter

**DOI:** 10.3390/ijms22010329

**Published:** 2020-12-30

**Authors:** Silvia Da Ros, Giulia Nicoletto, Riccardo Rigo, Silvia Ceschi, Eleonora Zorzan, Mauro Dacasto, Mery Giantin, Claudia Sissi

**Affiliations:** 1 Department of Pharmaceutical and Pharmacological Sciences, University of Padua, 35131 Padua, Italy; silviadaros31@gmail.com (S.D.R.); giulia.nicoletto.1@studenti.unipd.it (G.N.); riccardo.rigo.1@studenti.unipd.it (R.R.); silvia.ceschi@studenti.unipd.it (S.C.); 2 Department of Comparative Biomedicine and Food Science, University of Padua, 35020 Legnaro, Italy; eleonora.zorzan@studenti.unipd.it (E.Z.); mauro.dacasto@unipd.it (M.D.); 3 CRIBI Biotechnology Center (Centro di Ricerca Interdipartimentale per le Biotecnologie Innovative), University of Padua, 35131 Padua, Italy

**Keywords:** G-quadruplex, *KIT* promoter, SP1, gene expression

## Abstract

The regulation of conformational arrangements of gene promoters is a physiological mechanism that has been associated with the fine control of gene expression. Indeed, it can drive the time and the location for the selective recruitment of proteins of the transcriptional machinery. Here, we address this issue at the *KIT* proximal promoter where three G-quadruplex forming sites are present (kit1, kit2 and kit*). On this model, we focused on the interplay between G-quadruplex (G4) formation and SP1 recruitment. By site directed mutagenesis, we prepared a library of plasmids containing mutated sequences of the WT *KIT* promoter that systematically exploited different G4 formation attitudes and SP1 binding properties. Our transfection data showed that the three different G4 sites of the *KIT* promoter impact on SP1 binding and protein expression at different levels. Notably, kit2 and kit* structural features represent an on-off system for *KIT* expression through the recruitment of transcription factors. The use of two G4 binders further helps to address kit2-kit* as a reliable target for pharmacological intervention.

## 1. Introduction

*KIT* is a proto-oncogene highly relevant in human cancers [1,2]. Indeed, constitutive activation or overexpression of the c-kit protein supports the development, growth and sustainment of several tumors. Consistently, the use of tyrosine kinase inhibitors (i.e., imatinib, nilotinib) is a validated therapeutic approach for the treatment of c-kit dependent cancers [3]. Unfortunately, protein mutations are frequently observed, thus making the cancer resistant to the treatment. Alternative strategies currently under development are based on targeted gene silencing. Among them, the suppression of c-kit expression derived from a selective targeting of the gene promoter has been considered for its potential to overcome resistance due to protein mutations [4].

The *KIT* proximal promoter is quite peculiar. Indeed, it contains three G-rich domains (kit1, kit2 and kit*) that can stably fold into G-quadruplex (G4) structures in the presence of the physiological concentration of potassium cations [5,6,7]. The G4 arrangements are non-canonical secondary structures, where a core of G-tetrads (four guanines paired through Hoogsteen hydrogen bonds) stacks one over the other to form a stable tetrahelical structure [8]. Accumulating evidence suggests that these higher order arrangements might work as regulatory elements to control gene transcription [9,10]. In the case of *KIT*, the G4s formation at the promoter has been associated with a reduction in the corresponding coded protein expression [11,12]. The high-resolution structures of kit1, kit2 and kit* G4 have been solved [6,7,13,14,15,16], thus paving the way to a rational design of new ligands able to stabilize them and, ultimately, to silence the gene in the cell. Nevertheless, the pharmaceutical potential behind the targeting of this promoter region has not been fully elucidated yet. Indeed, the molecular mechanism linking the occurrence of non-canonical DNA structures at these sites to the reduction in gene expression is not completely dissected; moreover, it is still unclear which domain represents the most relevant G-rich site to target in order to realize a selective silencing of *KIT*.

The complexity of this issue is consistent with the fact that this promoter region can undergo multiple G4 structural rearrangements. For example, it was confirmed that in solution kit2 can fold into two main thermodynamically stable G4 structures. Noteworthy, thier folding pathways comprise kinetic intermediates whose half-life is comparable to the timescale of transcriptional processes [17]. Last but not least, the mutual interaction of the monomeric G4s forming at close positions (kit2 and kit*) was observed in solution [18]. In addition to the complexity of this picture, the insertion of these G-rich strands in a more complex nucleic acids framework (specifically a long double stranded system) further alters the overall structural equilibria and, likely, their cellular functions.

With the aim to punctually address this issue, Terenzi and coworkers considered several *KIT* proximal promoter sequences, where they mutated all guanines at each single G-rich domain (and at a combination of two and three G-rich sites) and studied them in solution and in cells by a dual luciferase assay [19]. This approach optimally prevented G4 formation, thus allowing the identification of sites where the DNA conformational shift from double strand to G4 can maximally impair the gene transcription. However, according to the proposed design, a suppression of gene expression can also derive from the concurrent modification of consensus sequences for the multiple transcription factors that bind this promoter region [12]. An interesting example is represented by SP1, a transcription factor that can drive *KIT* transcription either alone or in complex with other regulator factors (i.e., GATA-2) [20,21]. Within the G-rich region of the *KIT* promoter there is a validated SP1 consensus site (GGGGCGT) that overlaps with kit* [22,23]. It turns out that an extensive mutation of all guanines at this site can prevent its binding at the promoter irrespective of the DNA conformational features.

To get rid of this drawback, here we prepared a small library of sequences in which each single G-rich domain (kit1, kit2 and kit*) was made unable to fold into G4 by a limited number of point mutations (two for each sequence). These mutated sequences were also properly paired to obtain longer frames where one, two or all the three G4s cannot form. In addition, we specifically retained or abolished the SP1 binding site at kit* (Table 1). After the identification and characterization of all the mutants, we performed site-directed mutagenesis experiments in which the above mentioned libraries were introduced into a plasmid in which luciferase expression was under the control of the wild type (WT) *KIT* promoter sequence. The domain that resulted more relevant for gene transcription was finally considered as a target of two G4 binders (AQ1 and AN6, Figure S1) that were previously confirmed to bind the *KIT* promoter and to reduce c-kit expression in cells [11,24].

## 2. Results

### 2.1. Design and Selection of Mutated Sequences

The high-resolution structures of the monomeric G4 assumed by kit1, kit2 and kit* are available [6,7,13,14,15,16]. Although the comparison of multiple kit1 or kit2 structural data acquired under different conditions, either by NMR or X-ray, highlighted smooth differences, the guanines paired in G-tetrads are consistently identified. These latter represented the sites where we planned to introduce G-T mutations in order to impair G4 formation. In addition, for kit*, our aim was to prevent G4 formation by keeping the control of the SP1 binding. It was reported that on this sequence one single G-T mutation was sufficient to independently prevent the G4 formation or the binding of SP1 [23]. Thus, to limit the changes in the promoter sequence and to keep a conserved number of mutations at each G4 forming site, we introduced two G-T mutations at each G-rich site. 

As a first step, we ascertained the structural equilibria in solution of all the mutants by Circular Dichroism (CD) spectroscopy upon the addition of increasing KCl concentration as well as after an annealing step in the presence of the metal ion (Figure S2).

**Figure 1:**
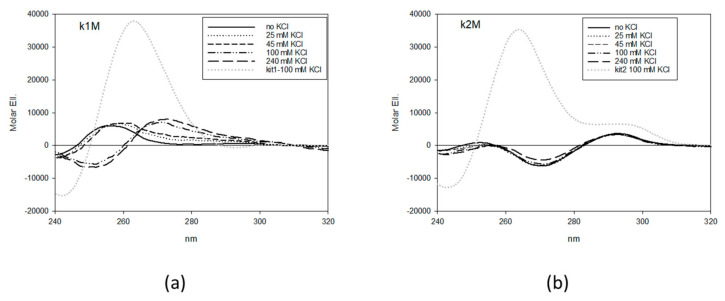
Circular dichroism titrations of 4 µM k1M (**a**) and k2M (**b**) with increasing concentrations of KCl in 10 mM Tris, pH 7.5, 25 °C. Grey dotted lines refer to the corresponding wild type sequences in 100 mM KCl.

In order to prevent G4 formation at kit1, the best results were obtained by working within the second G-run (G-T mutations at positions 7 and 8, k1M) whereas, in the case of kit2, G4 suppression required simultaneous changes at the first and the second G-runs (G-T mutations at positions 3 and 7, k2M) (Figure 1).

**Figure 2:**
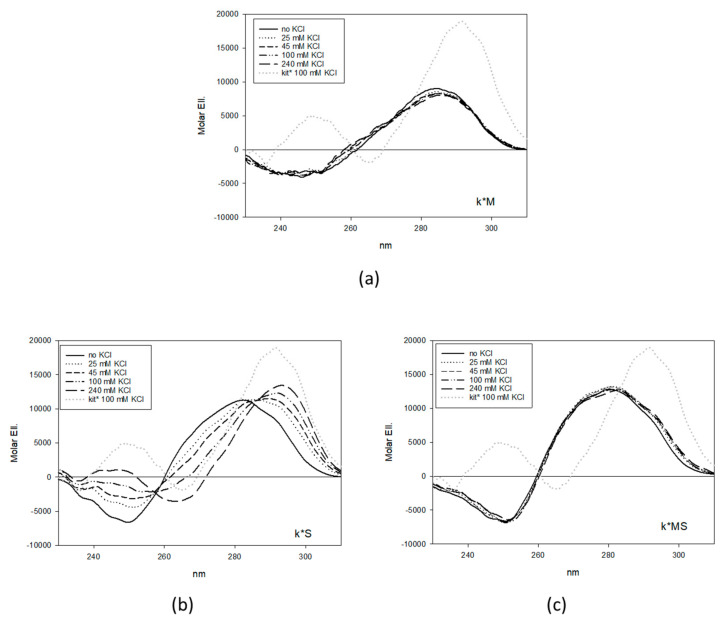
CD titrations of 4 µM k*M (**a**), k*S (**b**) and k*MS (**c**) with increasing concentrations of KCl in 10 mM Tris, pH 7.5, 25 °C. Grey dotted lines refer to the corresponding wild type sequences in 100 mM KCl.

As far as it concerns kit*, the consensus site of SP1 corresponds to the kit* central domain (residues 8–18). Thus, to abolish G4 folding while preserving SP1 binding, we mutated kit* at position 2 and 6 (k*M). As expected from previous studies [15], titration of this sequence up to 250 mM KCl showed no CD spectra changes related to G4 formation (Figure 2a). 

Conversely, to prevent the binding of SP1 by simultaneously suppressing or preserving the G4 folding, we focused on the second loop (residues 8–10, AGG) and the third G-run (residues 11–12, GG) (Figure 2b,c). Indeed, as reported by Kotar, G9 and 10 are not involved in G-tetrad formation [13]. Our data confirmed that when these two residues were mutated (k*S), the resulting oligonucleotide folded into an antiparallel G4 as the WT sequence, although with lower efficiency due to the removal of the stabilizing contribution provided by the G10-C18 base pair that works as a capping element for the G4 core. Conversely, by shifting the mutations at position 10 and 11 (k*MS), we suppressed G4 formation.

**Figure 3:**
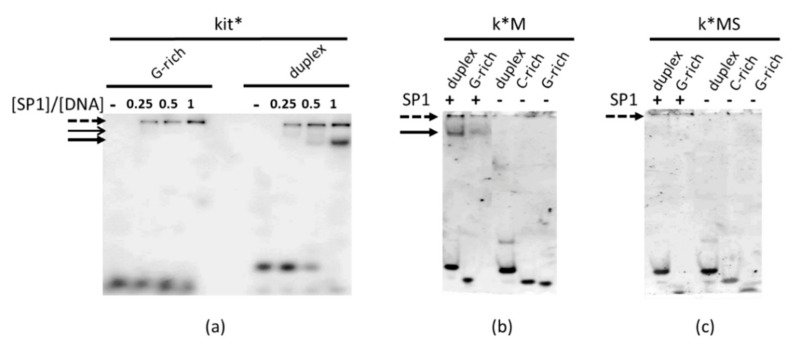
Electrophoretic mobility shift assay (EMSA) of 100 nM kit* (**a**) and related mutants (k*M (**b**) and k*MS (**c**)) in the presence of 0.25, 0.5 or 1 eq of SP1. Lanes labelled—refer to samples in the absence of protein; solid arrows point to DNA-protein complexes, the dotted one to not solved high molecular weight species.

To assess the SP1 binding properties of these kit*-derived mutants, we monitored their protein complex formation by electrophoretic mobility shift assay (EMSA) (Figure 3). In agreement with data from previous studies [23], both the single stranded and the ds form of kit* were able to bind the transcription factor. Nevertheless, the double stranded form of kit* appeared to be a best binder for SP1 in comparison to the G4 forming single stranded oligonucleotide. Indeed, in addition to the shared formation of not solved high molecular weight complexes (dotted arrow in Figure 3a), only with the double helix a clearly solved DNA-protein complex was detectable at 1:1 molar ratio (bold solid arrow in Figure 3a). According to our prediction, k*M still recognized the transcription factor and the complex formation was not fully suppressed under G4 promoting conditions. Conversely, k*MS did not bind SP1, either in the presence or absence of the complementary strand.

### 2.2. Site-Directed Mutagenesis and Transfection

**Figure 4:**
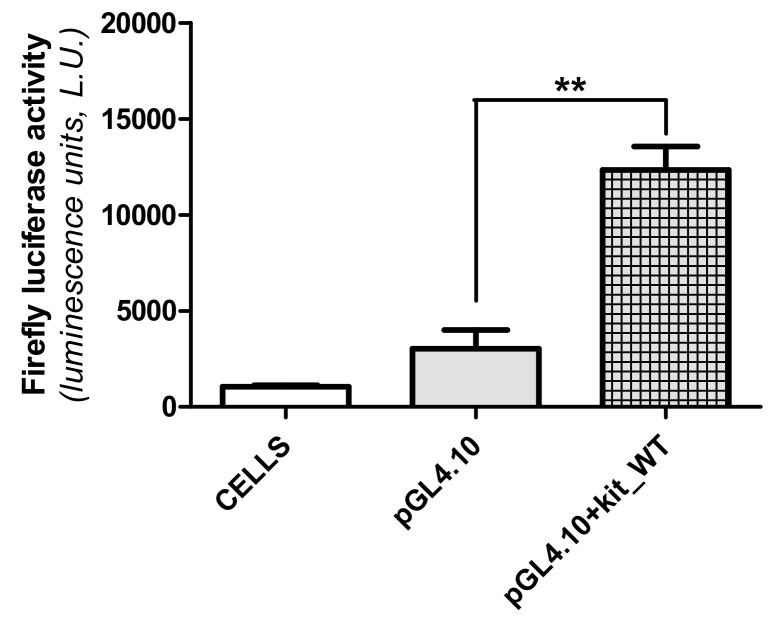
Transcriptional activity of the WT *KIT* proximal promoter. The promoter sequence (kit_WT: −65/−210 from ATG or −7/−152 from the TSS) was cloned into a pGL4.10 luciferase vector and transfected into MCF-7 cells. Firefly luciferase activity was assessed using ONE-GloTM Luciferase Assay System. pGL4.10+kit_WT transcriptional activity was compared to the one obtained for the empty pGL4.10 vector. Data (mean ± SEM) are representative of three independent experiments, each performed in triplicate. Statistical analysis: unpaired *t*-test (** *p* < 0.01).

The identified sequences represented a full library of possible structural and functional combination in terms of G4 folding and SP1 binding (Table 1). In order to assess their contribution to gene expression, we tested all of them by luciferase assays. To this purpose, first we inserted the WT sequence of the human *KIT* proximal promoter (kit_WT: −65/−210 from ATG or −7/−152 from the Transcription Starting Site (TSS), that includes the kit1, kit2 and kit* domains, into the multicloning site of a pGL4.10 [luc2] vector. This construct was used as a template for site-directed mutagenesis to obtain the plasmids corresponding to all the selected mutations. They were subsequently used to transfect MCF-7 cells and to monitor their contribution to the regulation of firefly luciferase expression. The WT sequence inserted into the plasmid was sufficient to significantly induce luciferase expression (*p* < 0.01, Figure 4).

**Figure 5:**
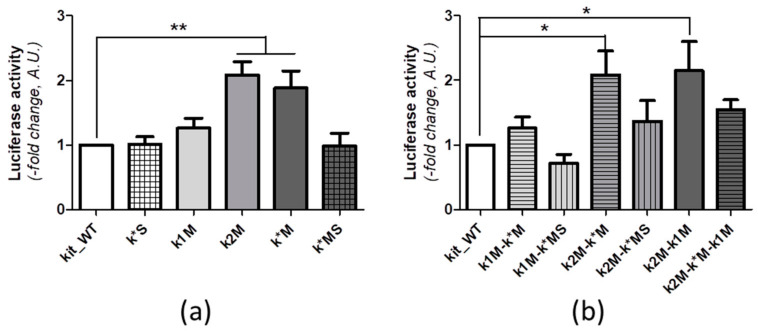
Luciferase activity in MCF-7 cells transiently transfected with plasmids containing kit_WT and mutated sequences at a single (**a**) or multiple (**b**) G-quadruplex (G4) forming sites. The firefly signal was normalized to renilla signal. The -fold change of the luciferase activity between WT and each mutated sequence was calculated in terms of arbitrary units. Five independent experiments were performed and each experimental condition was tested in sextuplicate. Data are expressed as means ± SEM. Statistical analysis: One way ANOVA followed by Dunnett’s multi comparison test (* *p* < 0.05; ** *p* < 0.01).

On this system, we moved to compare the effect of transcription upon the mutation of one single G-rich domain (Figure 5a). Distinctly from previously published data [19], the impairment of G4 formation at kit1 (k1M) did not significantly affect luciferase expression. Conversely, when we impaired G4 folding at kit2 or kit* (k2M and K*M), we observed a significant increment of luciferase activity in comparison to the WT sequence (*p* < 0.01), thus pointing to a major role exerted by kit2 and kit* G4 in the control of the promoter activity. Interestingly, whenever the recruitment of SP1 at kit* was suppressed (k*S and k*MS), the luciferase expression was comparable to the one obtained with the kit_WT sequence, irrespective of the G4 folding potential of kit*. These results suggest that whether the binding of SP1 at kit* appears to be required for the enhancement of gene expression observed in the absence of G-quadruplex folding, it is dispensable for the onset of gene expression. This picture fits with the confirmed presence of multiple transcription factor binding sites along the WT *KIT* promoter domain inserted in the plasmid that can support the basal expression of the protein [12,20,22]. 

In this context, the comparison of luciferase expression induced by plasmids simultaneously mutated at two or three G-rich sites further supports a fundamental role of kit2 folding in driving the transcription (Figure 5b). Indeed, only the concomitant mutation of kit2 and either kit* or kit1 (kit2M-k*M; kit2M-kit1M) showed a conserved increment of luciferase transcription (*p* < 0.05). In these cases, the protein expression was comparable to the one observed with the single-site mutated plasmid k2M. Nevertheless, whenever the SP1 binding at kit* was suppressed, we did not detect any increments in the protein production, thus suggesting a possible interplay between the two sites.

It is worth noting that the triple mutant k2M-k*M-k1M, as well as k*M-k1M, did not induce a significant increment of luciferase expression. This suggests that a more multifaceted structural (redistribution of guanines paired within G-tetrads [25,26]) and/or functional (altered recruitment of transcriptional machinery) relationship, that also involves kit1 in the interplay, cannot be excluded. 

### 2.3. SP1 Binding at kit2 and kit2-kit*

**Figure 6:**
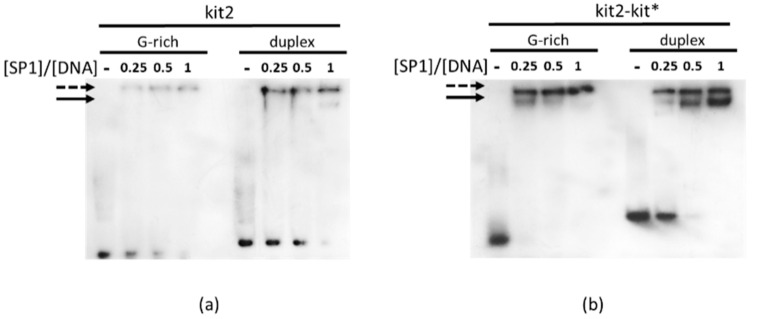
EMSA of 100 nM kit2 (**a**) and kit2-kit* (**b**) in the presence of 0.25, 0.5 or 1 eq of SP1. Lanes labelled—refer to samples in the absence of the protein; solid arrows point to DNA-protein complexes, the dotted one to not solved high molecular weight species.

Luciferase expression profiles indicated that the conformational features of kit2 and kit* played an active control on the transcriptional activity of *KIT*. As regards kit*, the increment of gene expression upon G4 disruption can be easily related to the greater affinity of SP1 for duplex vs. G4 DNA. A similar mechanism could be envisaged for kit2 as well, since a SP1 binding site overlapping the kit2 domain has already been predicted [27]. Here, we confirmed that the double stranded form of kit2 actually formed a solved complex with SP1, although with much lower efficiency in comparison to kit* (Figure 6). In agreement with the behavior observed with kit*, kit2–SP1 interaction was prevented by G4 formation.

Interestingly, EMSA confirmed that the proximal locations of kit2 and kit* further increment the protein recruitment. This was optimal on the duplex, whereas in the presence of the single G-rich strand, we were not able to dissect the formation of solved complexes. 

### 2.4. Binding of Candidate Ligands at kit* and kit2-kit*

**Figure 7:**
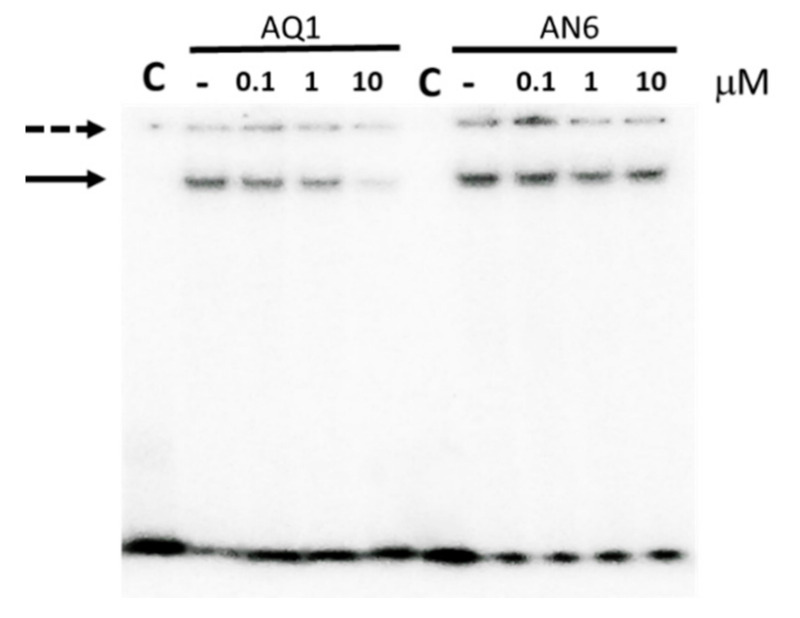
EMSA of 100 nM ds_kit*-SP1 (1:1 molar ratio) in the presence of increasing concentration of AQ1 or AN6. Lane C refers to samples in the absence of the protein; lanes labelled—refer to samples in the absence of ligand; solid arrows point to DNA-protein complexes, the dotted one to not solved high molecular weight species.

In previous works, we identified two promising G4 ligands (AQ1 and AN6, Figure S1) that reduced *KIT* expression in cancer cells by a direct recognition of its promoter [24]. The herein presented data pointed to kit* as the site where the ds-G4 conformational shift mainly influences SP1 binding. Thus, we tested whether our ligands can directly affect the protein-DNA complex formation. As shown in Figure 7, the addition of increasing AQ1 concentrations displaced SP1 from the DNA, whereas in the same concentration range AN6 was remarkably less efficient.

We previously reported that AQ1 was more cytotoxic than AN6 and we related this to its higher affinity for the G4 folded form of kit1 and kit2. However, whereas kit1 and kit2 fold into parallel G4s, kit* assumes an antiparallel G4 and the different topology can largely modulate the ligands recognition. Thus, to unambiguously correlate SP1 displacement to the DNA binding properties of our ligands, we further investigated their interaction with the G4 folded kit*.Surface Plasmon Resonance (SPR) data confirmed that AQ1 showed one-order magnitude higher affinity than AN6 also for the G4 assumed by kit* (Table 2). CD titrations provided a complex stoichiometric [ligand]/[G4] = 2/1 for both the ligands, thus supporting that both of them stacked on the external G-tetrads (Figure S3A,B). It is worth noting that the accessibility of the kit* external tetrads is different due to the contribution of accessory stabilizing interactions that are supported by residues involved in SP1 recognition (G10). Thus, to probe the ligands recognition at the two G4 sides, we substituted with the fluorescent analogue 2-aminopurine (AP), and the adenines at positions 5 and 8 that are located at the top and bottom tetrads, respectively (substituted sequence names kit*5AP and kit*8AP, respectively). Interestingly, both ligands did not discriminate between the top and bottom tetrads (Table 2).

Due to the suggested relevance of the longer kit2-kit* domain for the transcriptional control, we also analyzed the binding of our ligands on this sequence. The complex structural equilibria of this sequence in solution prevented a proper quantitative analysis of the binding. Nevertheless, CD titrations clearly indicated that the binding process was completed at a stoichiometric ratio of four ligands for each DNA strand (Figure S3C,D). Since this domain can fold into two interacting G4s, this result indicates that both ligands are able to break the G4-G4 interaction to obtain access to all the four external tetrads of the two G4 modules. 

## 3. Discussion

Despite the large number of studies focused on the consequences of G4 formation at the gene promoter on gene expression, a general model is not available yet. This lack reasonably reflects the huge variability of G4 structural features and the position at which they are forming in the proximal promoters (i.e., sense vs. antisense strand, distance from TSS, chromatin accessibility, epigenetic modifications) [9,28]. Moreover, it must be considered that the specific protein-DNA interaction pattern sensibly depends on DNA sequence and structure. This picture rationalizes the evidence that upon G4 formation some genes can be silenced, whereas others show an increment in transcription. 

As far as it concerns the *KIT* promoter, all currently available data point to a reduction in gene expression upon G4 formation. This correlation was derived by the use of G4 binders in principle designed to selectively target one G4 site of the promoter. However, tested compounds generally are not able to discriminate among different G4 structures so this approach fails to identify the contribution of each G4 forming site to the overall process. A closer look was provided by transfection studies with mutants at selective G-rich domains in the promoter [22,23]. However, to consider the more complex system that covers multiple G4 forming sites, reported works changed the primary sequence of the promoter to a large extent with concurrent consequences on the structural equilibria of the nucleic acid and on the protein recruitment [19].

Here, we showed that a very limited number of mutations are sufficient to prevent G4 formation at kit1, kit2 and kit*. This minimal variation in the overall sequence of the promoter is expected to preserve at least the large majority of the protein consensus sites herein located. As well, point mutations can be efficiently paired to mutually control G4 formation and transcription factor recruitment.

Our models indicated that G4 formation at kit2 and kit* greatly impacts on *KIT* expression. In addition, we showed that both kit2 and kit* bind SP1, although with different efficiency. This result perfectly matches with the reported critical role of this transcription factor in driving protein expression and with its concomitant suppression when all the guanines were mutated. Interestingly, despite a lower affinity of kit2 for SP1, its folding plays a relevant role in gene expression; this might underline the binding of other transcription factors at this site that grant a basal expression of the gene or, as previously suggested, that act in cis with other sites to support the basal *KIT* expression [22].

Unexpectedly, we did not detect any increment of protein expression upon the mutation of kit1, a result that poorly matches with previously reported transfection studies [19]. However, kit1 mutation reverts the incremented luciferase expression caused by impairing G4 formation at kit* or kit2-kit*. Consequently, we cannot fully exclude a modulation of kit1 on the transcriptional regulation exerted by kit2 and kit*. This model actually fit with the cellular effects detected upon treatment with G4 binders designed to target kit1 [4]. However, it is worth to remind ourselves that, despite the remarkable difference in the structural features of kit1, kit2 and kit*, we showed that stacking agents such as the herein tested AQ1 and AN6 poorly discriminate among them. On these bases, an intriguing possibility to realize a selective functional recognition of *KIT* is to move from the targeting of a single G4 unit towards the kit2-kit* higher order-system that appears to control transcription largely. Actually, our tested ligands target kit2-kit* as well as the isolated domains by virtue of its dynamic structural features [18]. Nevertheless, as recently supported by molecular dynamics studies [29], kit2-kit* represents a system with unique structural features that can be successfully exploited for innovative drug design projects.

## 4. Materials and Methods

Synthetic oligonucleotides and primers were provided by Biosense and used without further purification. 

The anthracene (AN6) and the anthracenedione (AQ1) derivatives were synthesized according to the reported procedures [30,31]. Ligands stock solutions (10 mM) were prepared in DMSO and freshly diluted in the required buffer.

### 4.1. CD Analysis

Circular dichroism spectra were recorded on a Jasco J-810 spectropolarimeter equipped with a Peltier temperature controller using 1–10 mm path-length cells in the 230–450 nm wavelength range. For each spectrum, 3 scans were acquired at a 50 nm/min scanning speed. DNA substrates (Kaneka Eurogentec S.A. Seraing, Belgium) were used at 4 µM final concentration. Before use, all DNA solutions were annealed in 10 mM Tris, pH 7.5. Titrations were performed at 25 °C by adding increasing concentrations of KCl. DNA titrations with ligands were performed in 10 mM Tris, 150 mM KCl, pH 7.5. Observed CD signals were converted into molar ellipticity [Θ] = deg × cm^2^ × dmol^−1^ (Mol. Ellip.) calculated by the DNA residue concentration in solution determined by UV absorbance at 260 nm.

### 4.2. Electrophoretic Mobility Shift Assay (EMSA)

Oligonucleotides were previously labelled with ^32^P at the 5′. Then, 100 nM oligonucleotide solutions were annealed o.n. in the presence (ds-samples) or absence (G4-samples) of an equimolar amount of the complementary strand. Afterwards, increasing concentrations of SP1 were added and after 1 h incubation in 10 mM TRIS, 150 mM KCl, pH 7.5 at 25 °C samples were loaded on a native 6% polyacrylamide gel (75:1 acrylamide: bisacrylamide) in 1X TBE (89 mM Tris, 89 mM boric acid and 2 mM EDTA).

### 4.3. Surface Plasmon Resonance (SPR)

Surface Plasmon Resonance measurements were performed on a Biocore X100 (GE Healthcare Life Sciences, Little Chalfont, UK) using a streptavidine-coated sensor chip functionalized with previously annealed 5′- biotinylated oligonucleotide until a 400 RU response. Sensorgrams were acquired using serial dilution of tested compounds (0–20 µM) and a constant final concentration of DMSO < 1% was present in all solutions. Compound solutions were injected at a 25 µL/min flow rate until a constant steady state was reached. After each run, a double regeneration was performed: 30 s with 10 mM glycine pH 7.5 followed by NaCl 1M and a 100 s stabilization period in running buffer. The experimental RU values recorded at the steady state were fitted according to one site saturation binding model + non-specific:

RU = ((RU_max_ *L)/(K_d_ + L)) + (n*L) (1)

where RU was the recorded resonance units at the steady state, RU_max_ was the maximal RU value, L was the concentration of ligand in the flushing solution and n was the non-specific interaction of the ligand with the chip.

### 4.4. Fluorescence

Fluorescence measurements were performed on a JASCO-FP-6500 spectrofluorometer equipped with a Peltier temperature controller. All spectra were acquired in 10 mM TRIS, 150 mM KCl, pH 7.5 at 25 °C using a quartz cell with a 10 mm optical path. Emission was acquired in the 320–600 nm range with the excitation wavelength fixed at 305 nm. kit*-5AP and kit*-8AP were previously annealed in the working buffer at a final concentration of 0.5 µM and titrated with increasing concentrations of ligands. Florescence data at 370 nm were fitted according to a single binding site model with the following equation:

F = (F_0_ *L)/(K_d_ + L) (2)

where F was the recorded signal 305, F_0_ was the signal in the absence of ligands and L was the concentration of ligand.

### 4.5. Site-Directed Mutagenesis

A 278 base pairs fragment, encompassing the proximal promoter and the 5′ flanking region of the human *KIT* oncogene and including the three G4 forming sequences, was initially amplified from whole blood genomic DNA using 5′-GCATTAACACGTCGAAAGAGC-3′ and 5′-GCAGAACGCAGAGAAAATCC-3′ as forward and reverse primers, respectively. The PCR product obtained from the first end-point PCR was then used as a template for a nested PCR, in which we used forward and reverse primers (F: 5′-AGCAGGtac/CAGACGCCGCCG-3′ and R: 5′-TGCAGCtAG/CGCGGCAAAGCC-3′) harboring the restriction sites (underlined sequence) for KpnI and NheI, respectively. The amplified product was purified from gel, digested with both enzymes (NEB, Euroclone, Milan, Italy) following the manufacturer’s instructions and subcloned, at the KpnI/NheI sites, into the reporter plasmid pGL4.10 expressing firefly luciferase (Promega, Madison, WI, USA). The obtained clones were sequenced to check for the correct insert ligation.

To selectively mutate the required sites, pGL4.10 + kit_WT plasmid was used as a template for site-directed mutagenesis with the In-Fusion HD Cloning kit (Clontech, Mountain View, CA, USA). Briefly, oligonucleotide primers (Table 3) were designed ex novo as per the manufacturer’s instructions.

The PCR amplification of the k2M and k2M-k1M sequences was performed using the Taq polymerase provided by the In-Fusion^®^ HD Cloning Kit and following the manufacturer’s instructions. Conversely, all the other mutations were obtained using Q5^®^ Hot Start High-Fidelity DNA Polymerase (NEB, Euroclone, Milan, Italy), and according to the following PCR conditions: an initial denaturation step at 98 °C for 30 s (hot start), 35 cycles of 5 or 10 s at 98 °C, 20 sec at 68 °C, 210 s at 72 °C, and a final extension step of 2 min at 72 °C. 

To re-close the plasmid, the In-Fusion reaction was assembled with each linear construct containing the mutation. Finally, 2.5 µL of the In-Fusion reaction was used to transform Stellar Competent Cells. At least five or ten colonies for each construct were isolated and sequenced with universal RV3 primer (BMR Genomics, PaduaS, Italy) to ascertain the insertion of the correct mutation. 

Plasmids containing the correct sequences were grown on a larger scale and purified with Plasmid Midi kit (Qiagen, Hilden, Germany).

### 4.6. Cell Culture

The breast adenocarcinoma human cell line MCF7 (Leibniz Institute DSMZ-German Collection of Microorganisms and Cell Cultures, Braunschweig, Germany) was maintained in T25 flasks under a humidified 5% CO_2_ atmosphere at 37 °C. Cells were grown in Eagle’s Minimal Essential Medium (EMEM, Gibco^®^ Life Technologies, Carlsbad, CA, USA) supplemented with 10% fetal bovine serum (Gibco^®^ Life Technologies, Gaithersburg, MD, USA), 2 mM L-glutamine (NEB, Euroclone, Milan, Italy), 1% non-essential amino acids (Euroclone), 1% penicillin/streptomycin (Euroclone) and Human insulin (10 μg/mL) (Elli Lilly & Co., Indianapolis, IN, USA). Cell number and viability were checked by using the Trypan Blue dye exclusion test (Sigma-Aldrich Co., St. Louis, MO, USA). For all the experiments, cells were used from passage 5 to passage 25 maximum. Furthermore, cell cultures were checked for Mycoplasma spp. contamination both before and at the end of experiments through PCR Mycoplasma Test Kit (PromoKine, Heidelberg, Germany). 

### 4.7. MCF-7 Transfection

MCF-7 cells were seeded at a density of 2 × 10^5^ cells/100 µL in a 96-well plate in Opti-MEM culture medium (Thermo Fisher Scientific, Waltham, MA, USA) with 5% FBS. After 24h, cells were transfected with 100 ng of plasmid DNA using TurboFect transfection reagent (Thermo Fisher Scientific, Waltham, MA, USA) at the ratio 2:1 of transfection reagent:DNA). In the case of co-transfection, 92 ng of pGL4.10 Firefly luciferase vector (containing the WT or mutated KIT promoter) and 8 ng of pGL4.74 Renilla luciferase control plasmid (Promega, Madison, WI, USA) were used (ratio 1:12.5). Twenty-four hours later, the transfection mixture was removed and cells were kept at 37 °C for a further 24 h with only EMEM medium. For the preliminary assessment of pGL4.10+kit_WT activity (three independent experiments in triplicate), ONE-GloTM Luciferase Assay System (Promega, Madison, WI, USA) was used. For the subsequent evaluation of the transcriptional activity of mutated promoter constructs, Firefly and Renilla luciferase were assayed using the Dual Luciferase Reporter Assay System (Promega, Madison, WI, USA). Relative luciferase activities were obtained calculating the ratio between Firefly and Renilla luciferase activities. Five independent experiments were performed and each experimental condition was tested in sextuplicate.

### 4.8. Statistical Analysis

Luciferase assay data were analyzed by unpaired t-test or one-way analysis of variance (ANOVA) followed by Dunnett’s multiple comparison test. All analyses were performed using GraphPad Prism 5.0 software (Graphpad, La Jolla, California). A *p* value < 0.05 was considered statistically significant. 

## Figures and Tables

**Table 1:** Wild type (WT) sequences and related mutated sequences selected for transfection studies.

Name	Sequence ^1^
kit1	5′-AGGGAGGGCGCTGGGAGGAGGG-3′
k1M	5′-AGGGAG**TT**CGCTGGGAGGAGGG-3′
kit2	5′-CGGGCGGGCGCGAGGGAGGGG-3′
k2M	5′-CG**T**GCG**T**GCGCGAGGGAGGGG-3′
kit*	5′-GGCGAGGAGGGGCGTGGCCGGC-3′
k*M	5′-G**T**CGA**T**GAGGGGCGTGGCCGGC-3′
k*S	5′-GGCGAGGA**TT**GGCGTGGCCGGC-3′
k*MS	5′-GGCGAGGAG**TT**GCGTGGCCGGC-3′
kit2-kit*	5′-CGGGCGGGCGCGAGGGAGGGGAGGCGAGGAGGGGCGTGGCCGGC-3′
kit2-kit*-kit1 ^2^	5′-CGGGCGGGCGCGAGGGAGGGGAGGCGAGGAGGGGCGTGGCCGGCG CGCAGAGGGAGGGCGCTGGGAGGAGGG-3′

^1^ underlined guanines are those involved in G-tetrads formation in the WT sequences as derived by high-resolution structures; residues in bold are those mutated in this study; ^2^ corresponds to the G-rich domain present in the WT *KIT* promoter.

**Table 2:** Apparent dissociation constants (K_d_, µM) of tested ligands for kit* determined by SPR or by fluorometric titration in 10 mM Tris, 150 mM KCl, pH 7.5, 25 °C.

Ligand	kit* ^1^	kit*5AP ^2^	kit*8AP ^2^
AN6	6.71 ± 1.26	2.24 ± 0.17	1.78 ± 0.15
AQ1	0.51 ± 0.09	0.32 ± 0.02	0.31 ± 0.02

^1^ data derived by SPR titrations, ^2^ data derived by fluorometric titrations.

**Table 3:** Primer pairs used for site-directed mutagenesis.

Mutations	Primer Pair ^1^	Oligonucleotide Sequence 5′-3′	Primer Design
k2M	F	ACCCGTGCGTGCGCGAGGGAGGGGAGGCGAGGA	ex novo
	R	CGCGCACGCACGGGTCTCGCTTCTTCCCGGCGG	ex novo
k1M	F	CGGCGCGCAGAGGGAGTTCGCTGGGAGGAGGGGCTGCT	ex novo
	R	GCGAACTCCCTCTGCGCGCCGGCCACGCCCCTCCTCGC	ex novo
k*M	F	GAGGGAGGGGAGTCGATGAGGGGCGTGGCCGGCG	ex novo
	R	ATCGACTCCCCTCCCTCGCGCCCGCCCGGGTCTC	ex novo
k*S	F	GGATTGGCGTGGCCGGCGCGCAGAGGGAG	ex novo
	R	CCACGCCAATCCTCGCCTCCCCTCCCTCGC	ex novo
k*MS	F	GGAGTTGCGTGGCCGGCGCGCAGAGGGAG	ex novo
	R	CCACGCAACTCCTCGCCTCCCCTCCCTCGC	ex novo
k2M-k*M	F	ACCCGTGCGTGCGCGAGGGAGGGGAGTCGATGA	ex novo
	R	CGCGCACGCACGGGTCTCGCTTCTTCCCGGCGG	k2M-R
k2M-k*MS	F	GGAGTTGCGTGGCCGGCGCGCAGAGGGAG	k*MS-F
	R	CCACGCAACTCCTCGCCTCCCCTCCCTCGC	k*MS-R
k1M-k*M	F	GAGGGAGGGGAGTCGATGAGGGGCGTGGCCGGCG	k*M-F
	R	ATCGACTCCCCTCCCTCGCGCCCGCCCGGGTCTC	k*M-R
k1M-k*MS	F	GGAGTTGCGTGGCCGGCGCGCAGAGGGAG	k*MS-F
	R	CCACGCAACTCCTCGCCTCCCCTCCCTCGC	k*MS-R
k2M-k1M	F	ACCCGTGCGTGCGCGAGGGAGGGGAGGCGAGGA	k2M-F
	R	CGCGCACGCACGGGTCTCGCTTCTTCCCGGCGG	k2M-R
k2M-k*M-k1M	F	ACCCGTGCGTGCGCGAGGGAGGGGAGTCGATGA	ex novo
	R	CGCGCACGCACGGGTCTCGCTTCTTCCCGGCGG	k2M-R

^1^ F = Forward; R = reverse.

## Data Availability

Data is contained within the article or supplementary material.

## Supplementary Materials

The following are available online at https://www.mdpi.com/1422-0067/22/1/329/s1, Figure S1: Chemical structures of G4 ligands used in this work; Figure S2: CD spectra of 4 µM k1M, k*M and k2M ((a), (b) and (c)) acquired in in 10 mM Tris, 100 mM KCl, pH 7.5, 25 °C before and after an anneling step. For comparison in panel (d) the same data are reported for a mutated sequence of kit2 (k2TT) that has been discharged along the selection process; Figure S3: CD titrations of 4 µM kit* ((a) and (b)) and kit2kit* ((c) and (d)) with increasing concentrations of AN6 ((a) and (c)) or AQ1 ((b) and (d)) in 10 mM Tris, 100 mM KCl, pH 7.5, 25 °C. Red lines refer to the oligonucleotides in the absence of ligand. The inset report the relative variation of the optical signal as a function of the [ligand]/[DNA] molar ratio.

## Abbreviations

G4	G-quadruplex
CD	Circular Dichroism
EMSA	Electrophoretic Mobility Shift Assay
SPR	Surface Plasmon Resonance
AP	2-aminopurine
