# Supplementary material for: G-Quadruplex Modulation of SP1 Functional Binding Sites at the KIT Proximal Promoter
Source: Int J Mol Sci. 2020 Dec 30;22(1):329. doi: 10.3390/ijms22010329 (PMC7795597; doi:10.3390/ijms22010329)
Supplement: Supplementary file 1 [file ijms-22-00329-s001.pdf]

International Journal of *Molecular Sciences*

MDPI

Supplementary Materials

G-Quadruplex Modulation of SP1 Functional Binding Sites at the *KIT* Proximal Promoter

Silvia Da Ros [1,†], Giulia Nicoletto [1], Riccardo Rigo [1], Silvia Ceschi [1,†], Eleonora Zorzan [2], Mauro Dacasto [2], Mery Giantin [2,*] and Claudia Sissi [1,3,*]

[1] Department of Pharmaceutical and Pharmacological Sciences, University of Padua, 35131 Padua, Italy; silviadaros31@gmail.com (S.D.R.); giulia.nicoletto.1@studenti.unipd.it (G.N.); riccardo.rigo.1@studenti.unipd.it (R.R.); silvia.ceschi@studenti.unipd.it (S.C.)

[2] Department of Comparative Biomedicine and Food Science, University of Padua, 35020 Legnaro, Italy; eleonora.zorzan@studenti.unipd.it (E.Z.); mauro.dacasto@unipd.it (M.D.)

[3] CRIBI Biotechnology Center (Centro di Ricerca Interdipartimentale per le Biotecnologie Innovative), University of Padua, 35131 Padua, Italy

* Correspondence: mery.giantin@unipd.it (M.G.); claudia.sissi@unipd.it (C.S.)

† Authors contributed equally.

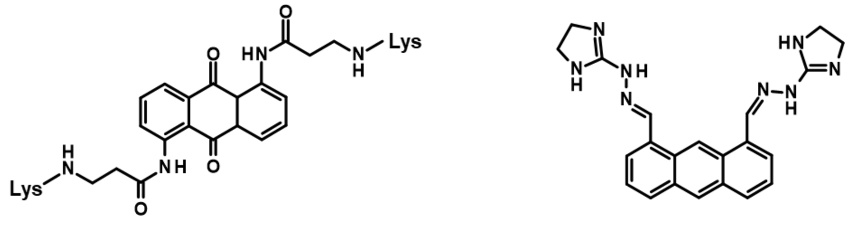

AQ1 AN6

Figure S1. Chemical structures of G4 ligands used in this work.

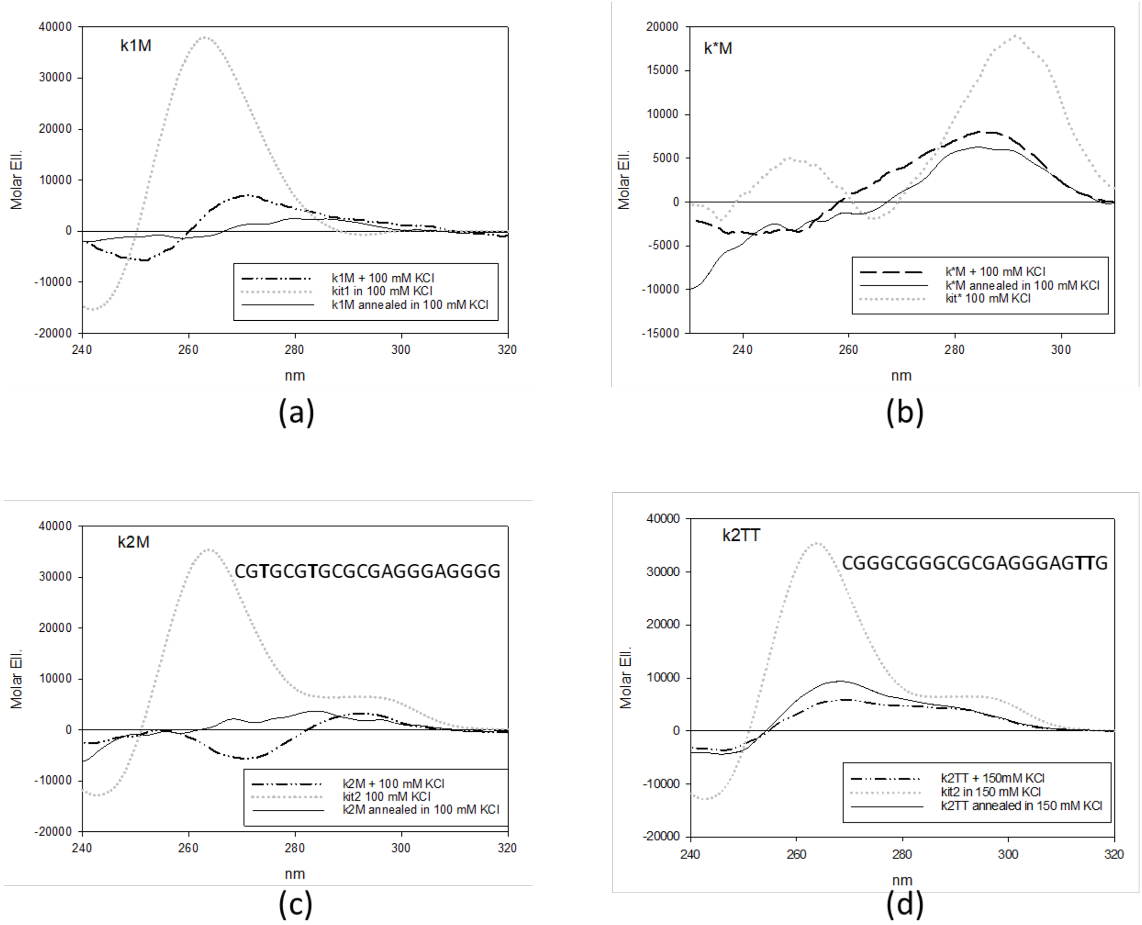

Figure S2. CD spectra of 4 µM k1M, k*M and k2M ((a), (b) and (c)) acquired in in 10 mM Tris, 100 mM KCl, pH 7.5, 25 °C before and after an anneling step. For comparison in panel (d) the same data are reported for a mutated sequence of kit2 (k2TT) that has been discharged along the selection process.

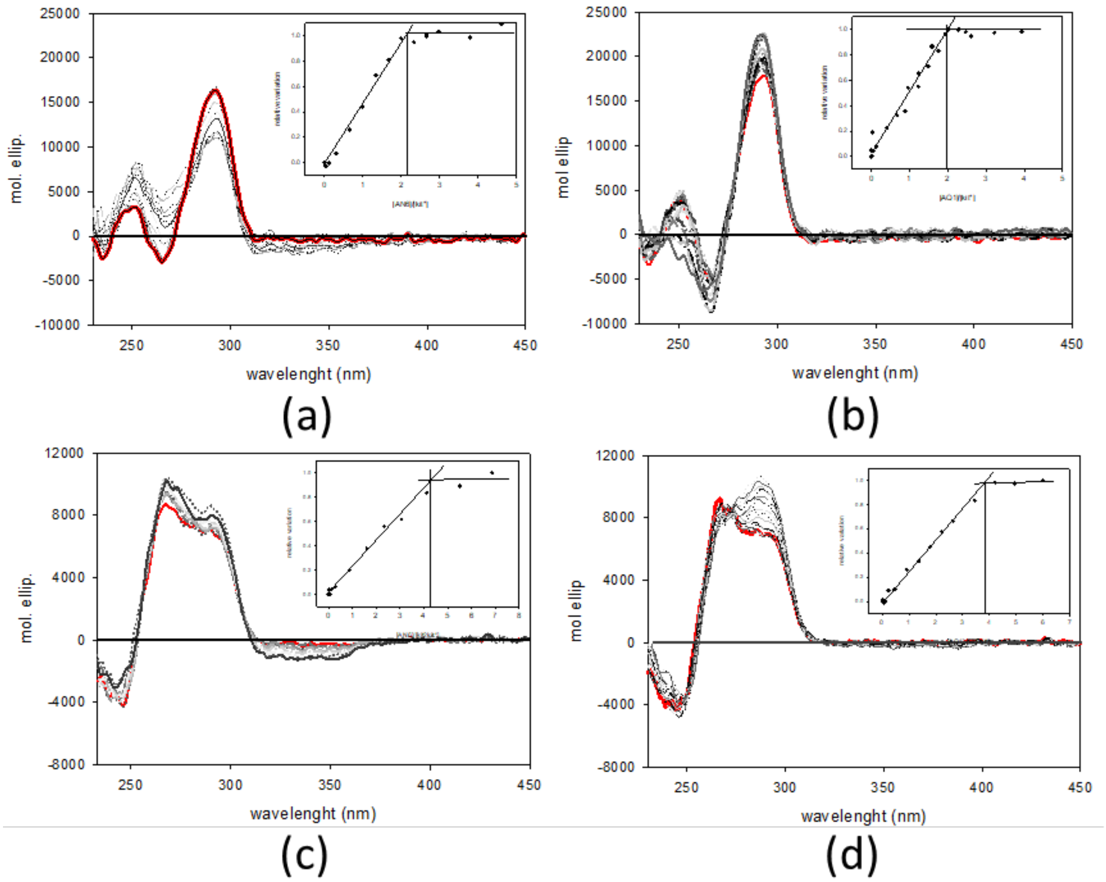

Figure S3. CD titrations of 4 μM kit* ((a) and (b)) and kit2kit* ((c) and (d)) with increasing concentrations of AN6 ((a) and (c)) or AQ1 ((b) and (d)) in 10 mM Tris, 100 mM KCl, pH 7.5, 25 °C. Red lines refer to the oligonucleotides in the absence of ligand. The inset report the relative variation of the optical signal as a function of the [ligand]/[DNA] molar ratio.
